# Prevalence and Risk Factors of Endometriosis Among Infertile Women in a Tertiary Care Center in South India

**DOI:** 10.7759/cureus.71772

**Published:** 2024-10-18

**Authors:** Nimmi Varghese, Indhumathi Shanmugam, Harini Sivamani, Anitha Durairaj

**Affiliations:** 1 Obstetrics and Gynaecology, Stoke Mandeville Hospital, Buckinghamshire Healthcare NHS Trust, Aylesbury, GBR; 2 Obstetrics and Gynaecology, Government Medical College, Thiruvananthapuram, Thiruvananthapuram, IND; 3 Obstetrics and Gynaecology, Chennai Urology and Robotics Institute Private Limited, Chennai, IND; 4 Obstetrics and Gynaecology, Dr. Aravind's IVF and Pregnancy Care, Madurai, IND

**Keywords:** dysmenorrhea, dyspareunia, endometriosis, infertility, prevalence, risk factors

## Abstract

Background

Endometriosis is a gynecological conundrum because of its challenging diagnosis and treatment. It seems to affect every aspect of a woman’s reproductive system, rendering her incapable of conceiving or achieving a successful pregnancy outcome independently. This study sought to determine the prevalence of endometriosis and clinical attributes of infertile women undergoing diagnostic laparoscopy.

Methodology

The current research is an analytical cross-sectional study that was carried out from April 2017 to December 2018. We included 103 women diagnosed with infertility and who underwent laparoscopy in the fertility center at Government Medical College Thiruvananthapuram, Kerala, a southern state of India. A semistructured questionnaire was used to collect information on the sociodemographic, menstrual, and reproductive characteristics. Family history and associated symptoms of endometriosis were elicited. Patients were put into four stages by the revised American Fertility Society (rAFS) grading, which was used for the laparoscopic staging.

Results

Among the 103 cases who underwent laparoscopy, 23 patients (22.3%) were diagnosed with endometriosis. Of the 23 cases of endometriosis, one (4.3%) had minimal endometriosis, two (8.7%) had mild endometriosis, and 10 (43.5%) each had moderate and severe endometriosis. Dysmenorrhea was significantly more prevalent among cases (87%, 20 participants) compared to controls (48.8%, 39 participants) (p = 0.001). Approximately 56.5% (13 participants) of cases had dyspareunia, compared to only 31.3% (25 participants) of controls, and this difference was statistically significant (p = 0.027). About 52.2% of the cases (12 participants) experienced premenstrual spotting, while none of the controls did. The p-value of 0.001 shows a statistically significant difference. Out of the total cases examined, 56.5% (13 participants) reached menarche at or before 12 years, whereas 43.5% (10 participants) attained it after reaching the age of 12. Among the control group, 31.2% (25 participants) of individuals had menarche at or before the age of 12, while 68.8% (55 participants) experienced it later. The difference that was observed had a statistically significant result (p = 0.027). The majority of cases (about 65.2%) experienced cycles lasting less than 21 days, with 30.4% having cycles within the normal range, and 4.3% experiencing cycles longer than 35 days. The significant difference in proportion is evident with a p-value of 0.015. Out of the cases, around 34.8% (eight participants) had a history of pelvic surgery. Compared to the control group, nearly 30.4% of the cases (seven participants) had a positive family history of endometriosis. There were no additional risk factors associated with the development of endometriosis.

Conclusion

The prevalence of endometriosis among infertile women undergoing diagnostic laparoscopy was more than one-fifth of the study patients. The common complaints were dysmenorrhea, followed by dyspareunia and premenstrual spotting. Risk factors associated with endometriosis were early menarche, frequent cycles, and positive family history.

## Introduction

The presence and proliferation of functional endometrial glands and stroma outside the uterine cavity define endometriosis. It is a chronic and recurrent disorder [1,2]. In 1860, von Rokitansky was the first to describe endometriosis as the presence of functioning tissues of the endometrium elsewhere than the uterine lumen [3]. Endometriotic implants may be situated throughout the pelvis, primarily affecting the ovaries, uterine ligaments, rectovaginal septum, and parietal peritoneum. Uncommon locations include the cervix, umbilicus, and laparotomy scars, as well as the pleural and pericardial cavities [4]. The clinical manifestation of endometriosis differs across women [5]. Patients often exhibit symptoms, including intermenstrual bleeding, dysmenorrhea, dyspareunia, dyschezia, and dysuria [6].

Endometriosis is classified as an epigenetic, hormone-regulated disorder characterized by progesterone resistance, whereas estrogen facilitates peri-lesional angiogenesis and neo-innervation, enabling the proliferation of endometriotic foci. Estrogens may diminish local immune surveillance by peritoneal fluid mononuclear cells and augment the proinflammatory microenvironment [7]. The exact mechanism by which endometriosis induces infertility requires evaluation. Many concepts have been put out to explain the etiology of endometriosis. An increase in total macrophages, leukocytes, and natural killer cells is seen in peritoneal fluid from women with endometriosis, in addition to higher concentrations of prostaglandins and inflammatory cytokines such as interleukin-1, interleukin-6, and vascular endothelial growth factor [8]. These modifications hurt sperm, oocyte, embryo, and fallopian tube functionality.

The precise prevalence cannot be readily determined since a conclusive diagnosis requires a laparoscopic evaluation. According to a systematic review, the global prevalence of endometriosis in women ranges from 6% to 10% [9]. A total of 10% to 15% of reproductive-age women are thought to have endometriosis, with a frequency of 70% among those who have continuous pelvic pain [5]. Endometriosis is present in 25%-50% of infertile females, whereas sterility is present in 30%-50% of women diagnosed with endometriosis. Endometriosis is six to eight times more common in infertile women than in fertile women [10].

For several years, researchers have disagreed on whether endometriosis causes infertility. Fecundity in a normal marriage is about 15% to 20%, while in endometriosis patients, it is closer to 2% to 10% [11]. There are a few hypotheses put forward to explain the link between endometriosis and infertility. It includes altered endometrial hormonal and cell-mediated mechanisms, abnormalities in ovulation and the endocrine system, and a skewed pelvic anatomy [12].

Imaging has limited efficacy in diagnosing endometriosis because of insufficient resolution for detecting adhesions or superficial peritoneal implants [13]. Laparoscopic exploration with biopsy of intraperitoneal cavity lesions is regarded as the gold standard diagnostic technique since it provides direct visualization of endometriosis lesions [14,15]. Established scoring methods for disease severity staging have a variable correlation with clinical symptoms or infertility. The updated American fertility score system is extensively used for illness staging. The American Society for Reproductive Medicine’s classification is used to document the amount and anatomical location of the illness [16].

Endometriosis affects almost 50% of infertile women who have surgical procedures. Infertile women are more likely to have endometriosis as the primary cause of their infertility, although little is known about the risk factors for this condition, despite its high occurrence and accompanying morbidity [17]. The correlation between endometriosis in infertile women and clinical symptoms is a multifaceted connection driven by several circumstances. Thus, the focus of this investigation was to determine the prevalence of endometriosis among infertile women undergoing diagnostic laparoscopy who were admitted to the tertiary care center in Kerala and to figure out the determinants that are associated with the presence of endometriosis among these women.

## Materials and methods

Study design, period, and participants

The current research is an analytical cross-sectional study that was carried out for a duration of one year and nine months (April 1, 2017, to December 31, 2018). We included women diagnosed with infertility who underwent laparoscopy in the fertility center at Government Medical College, Thiruvananthapuram, Kerala, a southern state of India.

Inclusion criteria

All patients with primary or secondary infertility who had diagnostic laparoscopy throughout the research period were included.

Exclusion criteria

Patients unwilling to take part in the trial were excluded.

Sample size and sampling technique

Mishra et al. conducted a hospital-based prospective investigation in which 502 women had laparoscopic examinations to evaluate the etiology of infertility; 276 (54.98%) were found to have endometriosis [18]. Using the formula N = 3.84 * p * q / d^2^, where p represents prevalence, q signifies the complement of p, and d shows the margin of error (with a 10% absolute error), we determined the sample size based on the aforementioned prevalence rate. The research requires a minimum of 96 samples. We included 103 infertile patients who were admitted to the Department of Obstetrics and Gynecology in a sequential order.

Data collection procedure

Data were gathered from the research participants via in-person interviews. Data was collected using a semistructured questionnaire. The questionnaire included sociodemographic variables, including age, education, socioeconomic level, body mass index (BMI), and smoking history. The menstrual history included age of menarche, monthly pattern, frequency, and length of menstrual flow. We gathered data on related symptoms, including dysmenorrhea, dyspareunia, chronic pelvic discomfort, premenstrual spotting, dysuria, diarrhea, and dyschezia. The reproductive history, including previous pregnancies, length and type of infertility, as well as oral contraceptive pill use, was obtained from patients. The family history of endometriosis and the frequency of coffee use were obtained. 

The laparoscopic assessment of endometriotic lesions exhibited a range of lesions. The dimensions, positioning, and intensity of the observed lesions were recorded to assess the severity of the condition. The mark was determined according to the graphic attributes of the lesion, with staging classified: values of 1-5 show minor illness, 6-15 imply mild disease, 16-40 reflect substantial disease, and values beyond 40 signal severe disease. The laparoscopic staging used the revised American Fertility Society (rAFS) grading, categorizing patients into four stages [16]. Pathologists made histological confirmation.

Ethical considerations

We received approval from the Institutional Ethics Committee of Government Medical College, Thiruvananthapuram, before the start of our study (approval number: 01/22/2017/MCT). A detailed explanation was provided to each participant, and their written consent was obtained prior to data collection.

Data analysis

All data were entered in MS Excel (Microsoft Corporation, Redmond, Washington, United States) and analyzed using IBM SPSS Statistics for Windows, Version 26 (Released 2019; IBM Corp., Armonk, New York, United States). Categorical variables are represented by frequency and percentage. We used the chi-square test and Fisher's exact test, when applicable, to evaluate the association between the prevalence of endometriosis and categorical factors. A p-value below 0.05 was deemed statistically significant.

## Results

Among the 103 cases who underwent laparoscopy, 23 patients (22.3%) were diagnosed with endometriosis. A total of 42 women (40%) with infertility had a fibroid uterus, while 11 patients were diagnosed with polycystic ovarian disease (PCOD), and 10 were diagnosed with pelvic inflammatory disease (PID). Table 1 shows the laparoscopic findings of the study samples.

**Table 1:** Distribution of laparoscopic findings of women with infertility PCOD: Polycystic ovarian disease; PID: pelvic inflammatory disease

Laparoscopic findings	Frequency	Percentage
Endometriosis	23	22.3
Fibroid	42	40
Congenital anomaly	5	4.8
Non-endometriotic ovarian cyst	8	7.8
PID	10	9.7
PCOD	11	10.6
Normal	4	3.8
Total	103	100

Table 2 shows the visual diagnosis of endometriosis. Of the 23 cases of endometriosis, one (4.3%) had minimal endometriosis, two (8.7%) with mild endometriosis, and 10 (43.5%) each with both moderate and severe endometriosis.

**Table 2:** Distribution of staging of endometriosis by visual diagnosis

Visual diagnosis of endometriosis	Frequency	Percentage
Minimal	1	4.3
Mild	2	8.7
Moderate	10	43.5
Severe	10	43.5
Total	23	100

In this study, we classified infertile women with endometriosis as cases (23) and infertile women without endometriosis as controls (80). Table 3 displays the basic characteristics of the study participants and their association with endometriosis. Of the total cases, 82.6% (19 patients) were individuals aged below 35, while 17.4% (4 patients) were individuals aged above 35. Based on the chi-square test, there is no statistically significant difference between the two age groups (p = 0.256). Among the total cases,, approximately 56.5% (13 patients) were graduates, while 17.4% (4 patients) had completed secondary school education, and 26.1% (6 patients) had completed primary school education. The difference in educational attainment did not show statistical significance (p = 0.454). Out of the total cases, the above poverty line (APL) group categorizes 65.2% (15 samples), while the below poverty line (BPL) group accounts for 34.8% (8 samples). Out of the total controls, the APL group accounts for 48.8% (39 samples), while the BPL group classifies the remaining 51.3% (41 samples). The observed difference in socioeconomic status did not reach statistical significance (p = 0.163). The examination of cases showed that 65.2% (15 samples) had a normal BMI, while 26.1% (6 samples) had a BMI of 25 kg/m^2^ or higher, and 8.7% (2 samples) had a BMI lower than 18.5 kg/m^2^. Out of the controls, 72.5% (58 samples) exhibited a normal BMI, while 23.8% (19 samples) had a BMI of 25 kg/m^2^ or higher, and 3.8% (3 samples) had a BMI below 18.5 kg/m^2^. The difference observed among BMI categories did not attain statistical significance (p = 0.585).

**Table 3:** Basic characteristics of the study participants and their relationship with endometriosis SES: Socioeconomic status; APL: above poverty line; BPL: below poverty line; BMI: body mass index

Basic characteristics		Endometriosis present		Endometriosis absent		Total		Fisher’s exact value	p-value
		Cases (23)	%	Controls (80)	%	n = 103	%		
Age in years	≤35	19	82.6	62	77.5	81	78.6	1.289	0.256
	>35	4	17.4	18	22.5	22	21.4		
Education	Primary school	6	26.1	16	20.0	22	21.4	2.619	0.454
	Secondary school	4	17.4	21	26.3	25	24.3		
	Graduate	13	56.5	38	47.5	51	49.5		
	Postgraduate	0	0.0	5	6.3	5	4.9		
SES	APL	15	65.2	39	48.8	54	52.4	1.942	0.163
	BPL	8	34.8	41	51.3	49	47.6		
BMI (kg/m^2^)	<18.5	2	8.7	3	3.8	5	4.9	1.074	0.584
	18.5-24.9	15	65.2	58	72.5	73	70.9		
	≥25	6	26.1	19	23.8	25	24.3		

Table 4 shows the symptoms of the study participants and their relationship with endometriosis. We observed dysmenorrhea in 87% of the cases (20 cases) and only 48.8% of the controls (39 individuals). We determined the difference between the two groups to be statistically significant (p = 0.001). A total of 7.8% of cases (11 samples) and 36.3% of controls (29 samples) reported pelvic pain. The statistical analysis revealed that the observed difference was not significant (p = 0.315). Dyspareunia was observed in 56.5% (13 patients) of cases, whereas only 31.3% (25 samples) of controls experienced this condition. The difference observed was of statistical significance (p = 0.027). Moreover, 52.2% of the cases (12 patients) showed premenstrual spotting. Among the controls, there were no instances of premenstrual spotting. The difference observed yielded statistical significance (p = 0.001). None of the endometriosis patients experienced dysuria or diarrhea. Six of the controls reported experiencing dysuria, and one reported experiencing diarrhea. An equal number of cases and controls reported catamenial pain. Based on the Fisher's exact test, there is no statistical significance (p > 0.05). 

**Table 4:** Symptoms of the study participants and their relationship with endometriosis *Fisher’s exact test was used

Symptoms associated with endometriosis		Endometriosis present		Endometriosis absent		Total		χ2	p-value
		Cases (23)	%	Controls (80)	%	n=103	%		
Dysmenorrhea	Yes	20	87.0	39	48.8	59	57.3	10.657*	0.001
	No	3	13.0	41	51.3	44	42.7		
Chronic pelvic pain	Yes	11	47.8	29	36.3	40	38.8	1.008	0.315
	No	12	52.2	51	63.8	63	61.2		
Dyspareunia	Yes	13	56.5	25	31.3	38	36.9	4.9	0.027
	No	10	43.5	55	68.8	65	63.1		
Premenstrual spotting	Yes	12	52.2	0	0.0	12	11.7	47.243*	0.001
	No	11	47.8	80	100.0	91	88.3		
Dysuria	Yes	0	0.0	6	7.5	6	5.8	1.832*	0.176
	No	23	100.0	74	92.5	97	94.2		
Diarrhea	Yes	0	0.0	1	1.3	1	1.0	0.29*	0.59
	No	23	100.0	79	98.8	102	99.0		
Catamenial chest pain	Yes	1	4.3	1	1.3	2	1.9	0.278*	0.598
	No	22	95.7	79	98.8	101	98.1		

Table 5 shows the study participants' risk factors and relationship with endometriosis. Out of the total cases examined, 56.5% (13 participants) reached menarche at or before 12 years, whereas 43.5% (10 participants) attained it after reaching the age of 12. Among the control group, 31.2% (25 participants) of individuals had menarche at or before the age of 12, while 68.8% (55 participants) experienced it later. The observed difference yielded a statistically significant result (p = 0.027). Among the cases, it was found that 73.9% (17 participants) have a menstrual flow lasting 2-7 days, whereas 26.1% (6 samples) have a menstrual flow lasting more than seven days. The observed difference did not reach statistical significance (p = 0.155). The majority of cases (about 65.2%) had periods that lasted less than 21 days, with 30.4% having cycles within the normal range and 4.3% experiencing cycles longer than 35 days. The significant difference in proportion is evident, as demonstrated by a p-value of 0.015.

**Table 5:** Risk factors in the study participants and their relationship with endometriosis *Fisher’s exact test was used

Risk factors associated with endometriosis		Endometriosis present		Endometriosis absent		Total		χ2	p-value
		Cases (23)	%	Controls (80)	%	n = 103	%		
Age at menarche	≤12 years	13	56.5	25	31.3	38	36.9	4.9	0.027
	>12 years	10	43.5	55	68.8	65	63.1		
Duration of menstrual flow	2-7 days	17	73.9	46	57.5	63	61.2	2.026	0.155
	>7 days	6	26.1	34	42.5	40	38.8		
Frequency of cycle	<21 days	15	65.2	26	32.5	41	39.8	8.424	0.015
	21-35 days	7	30.4	40	50.0	47	45.6		
	>35 days	1	4.3	14	17.5	15	14.6		
Type of infertility	Primary	19	82.6	65	81.3	84	81.6	0.022*	0.882
	Secondary	4	17.4	15	18.8	19	18.4		
Duration of infertility	<3 years	6	26.1	35	43.8	41	39.8	2.326	0.127
	>3 years	17	73.9	45	56.3	62	60.2		
Hormonal treatment	Yes	3	13.0	5	6.3	8	7.8	1.151*	0.283
	No	20	87.0	75	93.8	95	92.2		
History of pelvic surgery	Yes	8	34.8	1	1.3	8	7.8	31.198*	0.001
	No	15	65.2	79	98.8	95	92.2		
Family history of endometriosis	Yes	7	30.4	0	0.0	7	6.8	51.749*	0.001
	No	16	69.6	80	100.0	96	93.2		
Smoking	Yes	0	0.0	2	2.5	2	1.9	0.586*	0.444
	No	23	100.0	78	97.5	101	98.1		
Coffee intake	< 4 cups/ day	22	95.7	79	98.8	101	98.1	0.900	0.342
	≥ 4 cups/ day	1	4.3	1	1.3	2	1.9		

Further, 82.6% (19 participants) of the cases with endometriosis had primary infertility, while 17.4% (four participants) had secondary infertility. The observed difference did not show any statistical significance (p = 0.882). Out of all the cases, 73.9% (17 participants) had a duration of infertility exceeding three years, while 26.1% (six participants) had a duration of infertility below three years. The difference observed did not reach a statistical significance (p = 0.127). Out of all the cases, 87% (20 participants) had no documented history of hormonal treatment, whereas 13% (three participants) had a history of hormonal treatment. The observed difference was statistically not significant (p = 0.283).

Out of all the samples, 34.8% of cases (eight participants) had a previous record of pelvic surgery, while none of the controls had a history of pelvic surgery. The observed difference reached statistical significance (p = 0.001). Compared to the control group, nearly 30.4% of the cases (seven participants) had a positive family history of endometriosis. The observed difference was statistically significant (p = <0.001). None of the cases had a documented history of passive smoking, in contrast to two controls who did. The chi-square test did not show any statistical significance, with a p-value of 0.44.

## Discussion

Prevalence of endometriosis

The primary aim of this study was to determine the prevalence of endometriosis among infertile women who underwent laparoscopy. Among 103 patients who underwent laparoscopy, 23 patients were diagnosed with endometriosis, and the prevalence is 22.3%. According to a systematic review, the global prevalence of endometriosis in women ranges from 6% to 10% [9]. Endometriosis was shown to be 26.5% common among infertile women in a retrospective analysis of 1282 surgical patients conducted by Ashrafi et al. (2016) at an Iranian infertility institute [17]. Similar research by Khawaja in 2009 involved 796 women who underwent an exploratory laparoscopy with dye testing. A total of 134 women, or 16.8%, were diagnosed with endometriosis based on laparoscopic findings [19]. In a 2016 prospective study, Mishra et al. found that among infertile women who had laparoscopy, the prevalence was 54.98% [18]. This prevalence was higher compared to our study; the reason could be the disparity in the population studied and the large sample size used.

Relationship with demographic characteristics

Among the total of 103 patients who underwent laparoscopy, 23 samples were diagnosed with endometriosis, and the mean age in the endometriosis group was 28.96 years. The study by Mishra et al. on the prevalence of endometriosis among infertile women has a mean age of 28.55 ± 4.29, which is the same age group as in our study [20]. The average age of the study population in Khwaja et al.'s retrospective research was 29.3 years, and 796 of the women who were unable to conceive ultimately underwent diagnostic laparoscopy. Patients ranging in age from 25 to 33 made up the bulk of those cases [19].

Among the total cases, approximately 56.5% (13) were graduates, while 17.4% (four) had completed secondary school education, and 26.1% (six) had completed primary school education. The difference in educational attainment did not show statistical significance (p = 0.454). Endometriosis was not associated with a woman's level of education in a case-control study of infertile women conducted by Bérubé et al. [21]. Moen et al. found no noticeable relationship between endometriosis and educational attainment in their cross-sectional analysis of endometriosis risk variables [22]. In contrast, a case-control study by Moini et al. in Iran (2013) among 413 infertile women who underwent diagnostic laparoscopy documented that education was a predictive factor for severe endometriosis [23].

Nearly 65.2% of cases (15) had normal BMI, 26.1% (six) had BMI ≥ 25 kg/m^2^, and 8.7% (two) had BMI < 18.5 kg/m^2^. The observed difference between BMI categories was not statistically significant (p = 0.585). The same conclusion was noted in research by Moen et al. in Norway, which revealed no correlation between endometriosis and BMI [22]. There was also no relationship between BMI and endometriosis in a retrospective study by Hemming et al. that included 2,777 individuals [24]. Ashrafi et al. (2016) conducted retrospective research among 1,282 infertile women in Iran to identify risk factors of endometriosis. A total of 341 infertile women who had endometriosis identified by laparoscopy made up the research group (cases). The case and comparison groups did not vary significantly in terms of BMI according to the independent t test analysis [17].

Symptoms associated with endometriosis

We observed dysmenorrhea in 87% of the cases (20) and only 48.8% of the controls (39 individuals). We determined the difference between the two groups to be statistically significant (p = 0.001). Similar to our study, Bulletti et al. conducted a study on endometriosis and infertility, and dysmenorrhea was seen in 80% of cases [10]. Moderate to severe dysmenorrhea was linked to a two- to fivefold increased incidence of endometriosis, according to a research conducted by Calhaz-Jorge et al. on clinical prognostic variables for the condition in an infertile community of Portuguese women [25]. In a 2017 research on the clinical as well as laparoscopic aspects of endometriosis in infertile women, Mishra et al. found that dysmenorrhea was the most frequently reported symptom in 63.76% (176) of patients [18].

Moreover, 7.8% of cases (11) and 36.3% of controls (29) reported chronic pelvic pain. The statistical analysis revealed that the observed difference was not significant (p = 0.315). Mishra et al. found that 9.05% of endometriosis-related infertile women felt persistent pelvic discomfort [18]. The bulk of the clinical symptoms, physical exam results, and endometriosis staging were not associated with each other, according to research by Khawaja et al., with the exception of a thin built and limited uterine movement [19]. Our results are at odds with those of research by Calhaz-Jorge et al., which found that the incidence of modest to advanced endometriosis was double among women who had persistent pelvic discomfort [25]. The disparity could be because of a smaller sample size.

Dyspareunia was observed in 56.5% (13) of cases, whereas only 31.3% (25) of controls experienced this condition. The difference observed was of statistical significance (p = 0.027). A retrospective study of 1282 surgical patients by Ashrafi et al. in an infertility institute in Iran (2016) showed that 48.4% endometriosis patients had dyspareunia, and dyspareunia was significantly associated with endometriosis which is the same as in our study [23]. A study by Fedele et al. on the prevalence of symptoms in infertile women with endometriosis reported that dyspareunia was more prevalent regardless of the stage of disease [26]. A cross-sectional multicentric study by the Gruppo Italiano per lo Studio dell'Endometriosi stated that dyspareunia was reported in 56.8% of the patients with borderline significance [27]. These findings were comparable to our study findings.

None of the endometriosis patients experienced dysuria or diarrhea. Six of the controls reported experiencing dysuria, and one reported experiencing diarrhea. An equal number of cases and controls reported catamenial pain. Based on the Fisher's exact test, there is no statistical significance (p > 0.05). A study by Bulletti et al. in 2010 on endometriosis and infertility shows that altered bowel habits and dysuria are seen in only 1%-2% of the cases [10]. In this study, 52.2% of patients with endometriosis had premenstrual spotting. There was a significant association noted between endometriosis and premenstrual spotting (p < 0.001). Similar findings were reported by Ashrafi et al. as premenstrual spotting as a risk factor of endometriosis with (p < 0.05) [17].

Risk factors associated with endometriosis

Out of the total cases examined, 56.5% (13) reached menarche at or before 12 years, whereas 43.5% (10) attained it after reaching the age of 12. Among the control group, 31.2% (25) of individuals had menarche at or before the age of 12, while 68.8% (55) experienced it later. The observed difference yielded a statistically significant result (p = 0.027). The likelihood of endometriosis increases in women who have menarche at an early age. Consistent with our results, a cross-sectional investigation on endometriosis risk factors in infertile women found that menarche occurring before the age of 13 was significantly associated with endometriosis [22]. Additionally, Matalliotakis et al. found a correlation between early menarche and a higher incidence of endometriosis (p = 0.024), which is consistent with our findings [28]. Systemic review and meta-analysis by Nnoaham et al. in 2012 showed that there was an increased risk of endometriosis with early menarche [29].

Among the cases, it was found that 73.9% (17) have a menstrual flow lasting 2-7 days, whereas 26.1% (six) have a menstrual flow lasting more than seven days. The observed difference did not reach statistical significance (p = 0.155). A study by Calhaz-Jorge et al. on predictive factors of endometriosis among infertile women showed that there was no association between the duration of menstrual flow and endometriosis, and this finding is similar to our study’s finding [25].

The majority of cases (about 65.2%) had periods that lasted less than 21 days, with 30.4% having cycles within the normal range and 4.3% experiencing cycles longer than 35 days. The significant difference in proportion is evident, as demonstrated by a p-value of 0.015. The incidence of severe endometriosis was shown to be connected with a shorter cycle duration (p = 0.043), according to a 2013 research on indicators of endometriosis among infertile females conducted by Moini et al. [23]. Our study finding was comparable with the findings by Arumugam et al. [30], Matalliotakis et al. [28], Missmer et al., and Bérubé et al. [21].

Out of all the cases, 73.9% (17) had a duration of infertility exceeding three years, while 26.1% (six) had a duration of infertility below three years. The difference observed did not reach statistical significance (p = 0.127). Endometriosis frequency was not associated with the duration a woman had been infertile, according to a similar research by Bérubé et al. [21]. There was no statistically significant correlation between the duration a couple has been trying to conceive and the presence or absence of endometriosis, according to a retrospective analysis by Ashrafi et al. [17].

In this study, 82.6% (19) of the cases with endometriosis had primary infertility, while 17.4% (four) had secondary infertility. The observed difference did not show statistical significance (p = 0.882). A similar study by Matorras et al. on epidemiology of endometriosis among infertile women shows that parity was similar in endometriosis-associated infertile women and infertile women without endometriosis [31].

Out of all the cases, 87% (20) had no documented history of hormonal treatment, whereas 13% (three) had a history of hormonal treatment. The observed difference was statistically not significant (p = 0.283). Among infertile women, there is no statistically significant correlation between OCP usage and endometriosis risk, according to a 2016 research conducted by Ashrafi et al. [17]. 

Compared to the control group, nearly 30.4% of the cases (seven) had a positive family history of endometriosis. The observed difference was statistically significant (p = <0.001). Likewise, Ashrafi et al. discovered a strong relationship between endometriosis and a family history of the condition in their retrospective analysis (p < 0.001) [1].

Limitations

Given the limitations of resources, we used convenience sampling to carry out this research among infertility patients attending the tertiary care hospital in Kerala, South India. The results cannot be extrapolated to other parts of the nation because of the study's exclusive focus on individuals from a particular geographic area. Conducting multicentric research with a probability of sampling approach in Kerala might provide more positive findings. A further drawback of the current investigation was the reduced sample size. Restricting our case definition to those with laparoscopic confirmation of illness may result in our cases reflecting women with more severe conditions. The state of infertility may influence the impact of risk factors and should be evaluated meticulously. The relationships identified in this research may not be causative because of its cross-sectional design.

## Conclusions

Based on the findings, it was estimated that the prevalence of endometriosis among infertile women was more than one-fifth of the patients. The common complaints were dysmenorrhea, followed by dyspareunia and premenstrual spotting. The risk factors associated with endometriosis were early menarche, frequent cycles, and positive family history. Endometriosis in infertile women is prevalent and is becoming progressively identified because of the enhanced use of diagnostic techniques, such as laparoscopy, in infertility assessment. This research underscores the elevated frequency of endometriosis within our demographic, especially among asymptomatic infertile women. We urge the assessment and treatment of a patient presenting in the gynecological unit with the aforementioned history and symptoms, showing a strong suspicion of endometriosis.

## Figures and Tables

**Figure 1:**
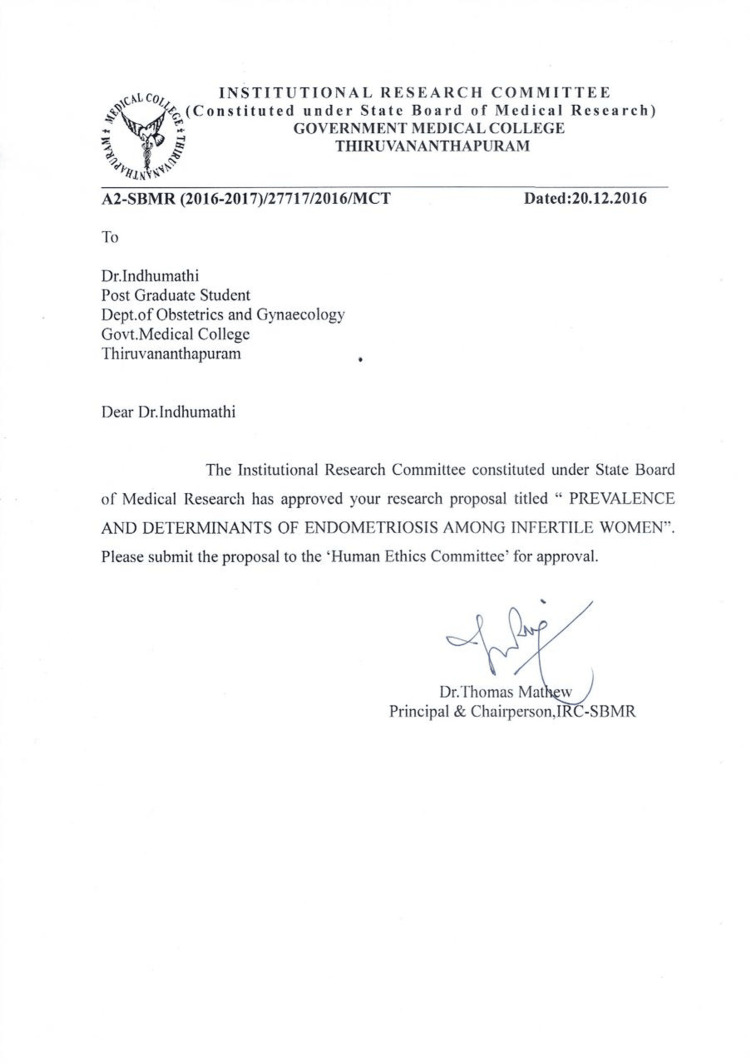
The Institutional Review Board (IRB) approval letter

## References

[1] Smolarz B, Szyłło K, Romanowicz H (2021). Endometriosis: epidemiology, classification, pathogenesis, treatment and genetics (review of literature). Int J Mol Sci.

[2] Tsamantioti ES, Mahdy H (2024). Endometriosis. http://www.ncbi.nlm.nih.gov/books/NBK567777/.

[3] Sourial S, Tempest N, Hapangama DK (2014). Theories on the pathogenesis of endometriosis. Int J Reprod Med.

[4] Carter JE (1994). Combined hysteroscopic and laparoscopic findings in patients with chronic pelvic pain. J Am Assoc Gynecol Laparosc.

[5] Parasar P, Ozcan P, Terry KL (2017). Endometriosis: epidemiology, diagnosis and clinical management. Curr Obstet Gynecol Rep.

[6] Sinaii N, Plumb K, Cotton L, Lambert A, Kennedy S, Zondervan K, Stratton P (2007). Differences in characteristics among 1,000 women with endometriosis based on extent of disease. Fertil Steril.

[7] Dmowski WP, Gebel HM, Braun DP (1994). The role of cell-mediated immunity in pathogenesis of endometriosis. Acta Obstet Gynecol Scand Suppl.

[8] Hill JA, Faris HMP, Schiff I, Anderson DJ (1988). Characterization of leukocyte subpopulations in the peritoneal fluid of women with endometriosis. Fertil Steril.

[9] Moradi Y, Shams-Beyranvand M, Khateri S (2021). A systematic review on the prevalence of endometriosis in women. Indian J Med Res.

[10] Bulletti C, Coccia ME, Battistoni S, Borini A (2010). Endometriosis and infertility. J Assist Reprod Genet.

[11] The Practice Committee of the American Society for Reproductive Medicine (2004). Endometriosis and infertility. Fertil Steril.

[12] Buyalos RP, Agarwal SK (2000). Endometriosis-associated infertility. Curr Opin Obstet Gynecol.

[13] Hsu AL, Khachikyan I, Stratton P (2010). Invasive and noninvasive methods for the diagnosis of endometriosis. Clin Obstet Gynecol.

[14] Baușic A, Coroleucă C, Coroleucă C (2022). Transvaginal ultrasound vs. magnetic resonance imaging (MRI) value in endometriosis diagnosis. Diagnostics (Basel).

[15] Zondervan KT, Becker CM, Missmer SA (2020). Endometriosis. N Engl J Med.

[16] The American Fertility Society (1985). Revised American Fertility Society classification of endometriosis: 1985. Fertil Steril.

[17] Ashrafi M, Sadatmahalleh SJ, Akhoond MR, Talebi M (2016). Evaluation of risk factors associated with endometriosis in infertile women. Int J Fertil Steril.

[18] Mishra VV, Bandwal P, Agarwal R, Aggarwal R (2017). Prevalence, clinical and laparoscopic features of endometriosis among infertile women. J Obstet Gynaecol India.

[19] Khawaja UB, Khawaja AA, Gowani SA (2009). Frequency of endometriosis among infertile women and association of clinical signs and symptoms with the laparoscopic staging of endometriosis. JPMA J Pak Med Assoc.

[20] Mishra VV, Gaddagi RA, Aggarwal R, Choudhary S, Sharma U, Patel U (2015). Prevalence; characteristics and management of endometriosis amongst infertile women: a one year retrospective study. J Clin Diagn Res.

[21] Gordon I, Boffetta P, Demers PA (1998). A case study comparing a meta-analysis and a pooled analysis of studies of sinonasal cancer among wood workers. Epidemiology.

[22] Moen MH, Muus KM (1991). Endometriosis in pregnant and non-pregnant women at tubal sterilization. Hum Reprod.

[23] Moini A, Malekzadeh F, Amirchaghmaghi E, Kashfi F, Akhoond MR, Saei M, Mirbolok MH (2013). Risk factors associated with endometriosis among infertile Iranian women. Arch Med Sci.

[24] Hemmings R, Rivard M, Olive DL, Poliquin-Fleury J, Gagné D, Hugo P, Gosselin D (2004). Evaluation of risk factors associated with endometriosis. Fertil Steril.

[25] Calhaz-Jorge C, Mol BW, Nunes J, Costa AP (2004). Clinical predictive factors for endometriosis in a Portuguese infertile population. Hum Reprod.

[26] Fedele L, Bianchi S, Bocciolone L, Di Nola G, Parazzini F (1992). Pain symptoms associated with endometriosis. Obstet Gynecol.

[27] Gruppo Italiano per lo Studio dell'Endometriosi (2001). Relationship between stage, site and morphological characteristics of pelvic endometriosis and pain. Hum Reprod.

[28] Matalliotakis M, Goulielmos GN, Matalliotaki C, Trivli A, Matalliotakis I, Arici A (2017). Endometriosis in adolescent and young girls: report on a series of 55 cases. J Pediatr Adolesc Gynecol.

[29] Nnoaham KE, Webster P, Kumbang J, Kennedy SH, Zondervan KT (2012). Is early age at menarche a risk factor for endometriosis? A systematic review and meta-analysis of case-control studies. Fertil Steril.

[30] Arumugam K, Lim JM (1997). Menstrual characteristics associated with endometriosis. Br J Obstet Gynaecol.

[31] Matorras R, Rodíquez F, Pijoan JI, Ramón O, De Terán GG, Rodríguez-Escudero F (1995). Epidemiology of endometriosis in infertile women. Fertil Steril.

